# Biodiversity–livestock interface: a case study

**DOI:** 10.1093/af/vfad068

**Published:** 2024-02-14

**Authors:** Patricia Barroso, Stefania Zanet

**Affiliations:** Department of Veterinary Sciences, Faculty of Veterinary, University of Turin, Grugliasco, Turin, Italy; Department of Veterinary Sciences, Faculty of Veterinary, University of Turin, Grugliasco, Turin, Italy

**Keywords:** conflicts, conservation, sustainable agriculture, wildlife-livestock coexistence, win–win outcomes

**Implications**

- The protection of environmental and ecological resources and commercial livestock management should not be a contradiction. Although the impact of livestock on wildlife conservation is under debate, it depends on factors such as climate, animal species, and grazing intensity. Eliminating livestock farming is not considered the solution since although it partly contributes to the problem, it also can play a part in the solution participating in the creation and maintenance of the multiple circular flows of nutrients in the soil, water bodies, and suitable conditions for certain wild species. In a pilot monitoring study in mainland Spain, we observed a positive relationship between livestock presence and diversity indexes.
- There is a strong linkage between biodiversity and livestock production and win-win outcomes are plausible. Landowners should be facilitated and incentivized, encouraging their involvement in enhancing biodiversity since they are the greatest managers of wildlife. Designing agroecosystems for optimizing and sustaining biodiversity and livestock productivity in the long term is challenging but necessary to reach socially sustainable rural development.

## Background

Livestock grazing is an important and widespread practice which covers 25% of the global land surface encompassing a wide variety of habitats from semiarid and arid regions to tropical and temperate territories (FAO, 2023). Livestock grazing usually occurs on rangelands, i.e., open areas of forage plants where domestic livestock cohabits with wildlife (Neilly et al., 2016). Grazing may lead to changes in the composition, assemblage structure, and functioning of ecosystems, for example, by ecological cascade effects on prey–predator dynamics or competition (Pafilis et al., 2013). These impacts can ultimately result in a diversity loss of sympatric wild species (Laurance et al., 2014). Agriculture development and overexploitation of certain habitats have been recognized as major drivers of biodiversity loss (Kok et al., 2020). Specifically, on a global scale, intensive livestock grazing has been implicated in the decline of vertebrate species richness, diversity and abundance as well as the loss of insect biomass (Mysterud et al., 2005; Wazna, 2016). This occurs through an alteration of both the biotic and abiotic elements of ecosystems caused mainly by the cumulative effect of habitat destruction of herbivory (changes in vegetation structure) and trampling (soil compaction and eutrophication) as well as the grazing management practices. However, the impact of livestock grazing on wildlife conservation depends on the agricultural practices since moderate levels of grazing could be harmless or even beneficial to wildlife, for example, when comparing abandonment/no grazing with extensive grazing (Aceto et al., 2004; Neilly et al., 2016; Pozo et al., 2021). Thus, deserting livestock farming is not considered the solution since although it partly contributes to the problem (land erosion, wastage of water, depletion of resources, disruption of nutrient cycles and eutrophication, biodiversity losses, etc.), it also can play a part in the solution participating in the creation and maintenance of the multiple circular flows of nutrients in the soil, water bodies and atmosphere, as has been illustrated for alpine ecosystems (Barbero-Palacios et al., 2023). Consequently, the protection of environmental and ecological resources and commercial livestock management should not be a contradiction. In this regard, sustainable livestock regimes which encompass environmental protection, animal welfare, biodiversity, food security and socio-economic promotion could become a way to balance food production and conservation without affecting rural development. In this way, it would be possible to achieve positive economic outcomes for the animal production sector while preserving the ecological processes that support biodiversity (Neilly et al., 2016; Kok et al., 2020). However, these actions are not always easy to apply depending on the context nor the desired results are obtained. For example, in Europe, several measures have recently been applied as part of the Common Agricultural Policy to increase biodiversity while preserving and supporting traditional agriculture, but biodiversity continues to decline (Kok et al., 2020).

In this paper, we briefly review the main positive and negative effects of the coexistence of livestock farming and biodiversity and present a case study from Spain evaluating the wild vertebrate species richness as a proxy of biodiversity in areas depending on the livestock presence.

## The debate on the biodiversity–livestock interface and factors determining its outputs

Despite rangelands being primarily used for livestock production, these habitats are suitable for wildlife, even outside protected areas, and their importance for biodiversity conservation cannot be overlooked (Neilly et al., 2016). In these environments, livestock and wildlife overlap with the subsequent consequences in both directions. For this reason, the biodiversity–livestock interface has led to much debate although relatively scarce literature (Laurance et al., 2014; Neilly et al., 2016; Kok et al., 2020).

Traditionally, a negative impact of livestock grazing on biodiversity (specifically plant, bird, and insect abundance and species richness) has been expected (Barzan et al., 2021). However, previous studies suggested that this relationship depends on numerous factors such as the wild species under study, the resident species assemblage, the grazing intensity, or climate. Conspicuous literature exists evaluating the effects of livestock on biodiversity in extreme contexts of grazing (intensive vs. traditional). However, studies on intermediate stocking rates and grazing pressure with rotation of herds are scarce (Neilly et al., 2016). Indeed, these breeding systems seem to be associated with an improvement of habitats for some wild vertebrates, potentially enhancing biodiversity conservation (Pozo et al., 2021). In addition, poor attention has been paid in the literature to factors determining the outputs of this relationship. For example, traditional pastoral systems in Africa and Central Asia have been compatible and even beneficial to the diversity of wild vertebrates and insects for many years without habitat degradation (Riginos et al., 2012).

Finally, to reliably assess the biodiversity-livestock relationship it is necessary to consider not only a wide range of biodiversity indicators but also the specific compositional changes and the responses of individual species to grazing (Riginos et al., 2012; Kok et al., 2020).

## Effects of the wildlife-livestock coexistence and consequences for biodiversity

**Figure 1.**
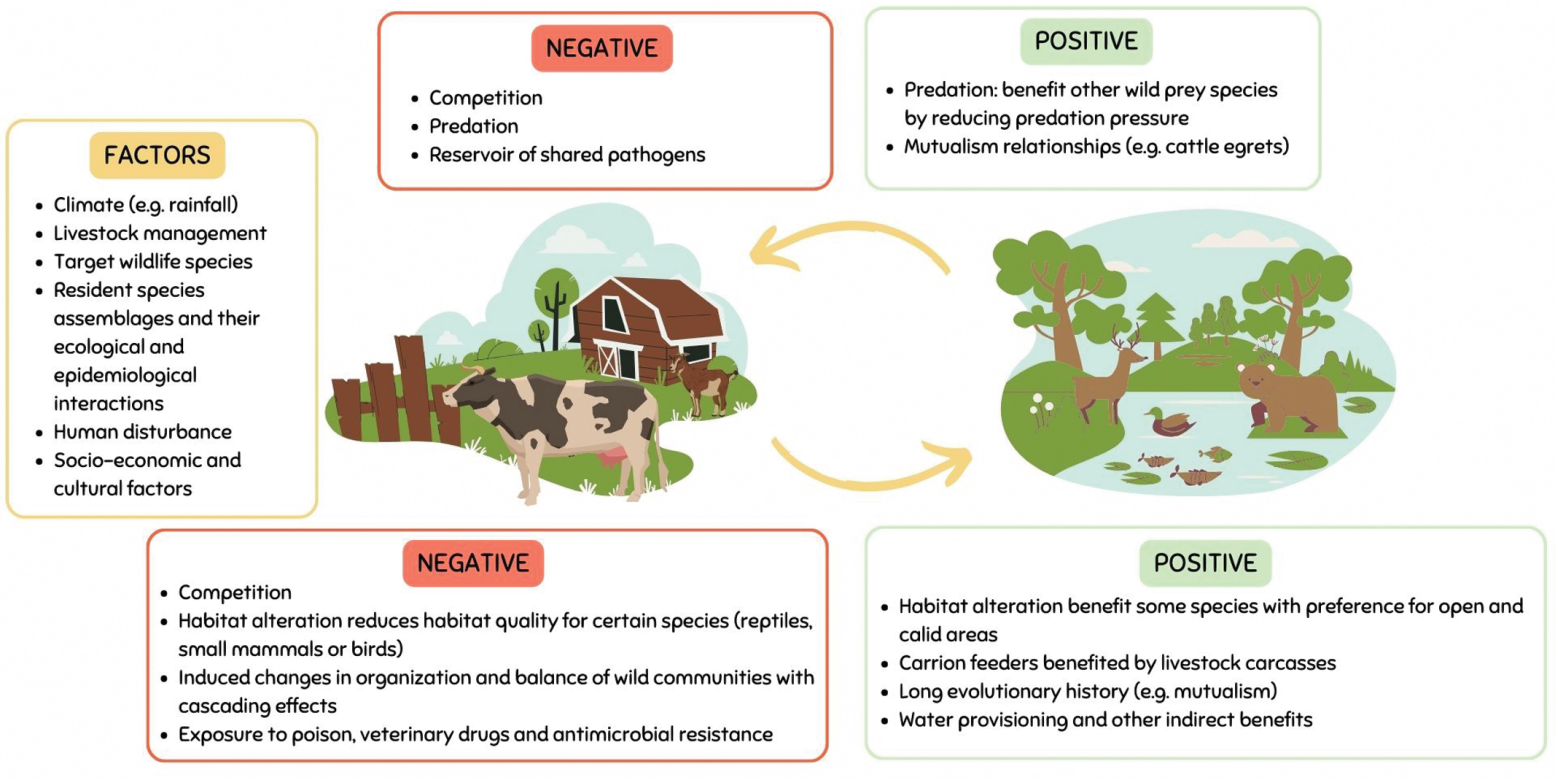
Main positive and negative effects of wildlife on livestock (top) and livestock on wildlife (bottom) derived from their coexistence, as well as the main factors which could determine these outputs.

Figure 1 displays the main positive and negative effects for livestock and wildlife derived from their coexistence, as well as the main factors which could determine these outputs.

### Wildlife to livestock

#### Negative

- Competition by consuming forage resources, altering livestock behavior and reducing its productivity (Riginos et al., 2012).
- Predation of livestock species by wild predators leading to economic losses (Neilly et al., 2016).
- Wildlife is a major reservoir of pathogens shared with livestock (Barroso et al., 2020).

#### Positive

- The predation of livestock species by wild predators can indirectly benefit other wildlife species since the predation pressure disappeared because of a perceived threat to livestock (Neilly et al., 2016; Pozo et al., 2021).
- There are mutualistic relationships between certain wild and livestock species such as the cattle egret (*Bubulcus ibis*) and cattle. In this case, cattle egrets serve as important ectoparasite biological control by feeding on the ectoparasites found on cattle and buffaloes.

### Livestock to wildlife

#### Negative

- Habitat alteration by grazing, trampling, and eutrophication. Changes in vegetation structure and trampling reduce habitat quality for certain species, thus potentially impacting the biodiversity. For example, some ground-nesting birds, ground-dwelling mammals, or some burrowing reptiles depend on complex ground habitats for survival, breed success or predation avoidance (Amar et al., 2011; Neilly et al., 2016).
- Competition by consuming forage resources (Riginos et al., 2012; Schieltz andand Rubenstein, 2016).
- Induced changes in the organization and balance of wild communities as well as in habitat structure, with some cascading effects (Pafilis et al., 2013; Wazna, 2016).
- Exposure to poison, veterinary drugs, and antimicrobial resistance (Neilly et al., 2016; Kok et al., 2020).

#### Positive

- Habitat alteration can also represent a benefit for those species with a preference for more open and grass-dominated habitats facilitating feeding and predation success. For example, raptors, some reptiles, mountain ungulates (chamois *Rupicapra rupicapra* or ibex *Capra ibex*) and Alpine marmots (*Marmota marmota*) (Huaranca et al., 2022).
- Carrion feeders are benefited from an increase in carcass availability (Neilly et al., 2016; Kok et al., 2020).
- Species with a long evolutionary history with grazing livestock species: for example, parasitic cowbirds (mutualism) or disturbance-tolerant birds.
- Indirect beneficial effects of associated land management practices such as the artificial provisioning of water, nightly housing of livestock or fires that can improve the grass quality (Riginos et al., 2012).

Even under high stocking densities, some vertebrate species are facilitated or not affected by intense grazing. As exemplified by the reptiles from arid zones of Australia and North America that do not respond to grazing (resilience) or arboreal reptiles that benefit from intense grazing activities (Kutt and Fisher, 2011). Another example, low-intensity grazing without clearing benefits the bird abundance and richness in Canada, Kenya, and Scotland (Huaranca et al., 2022).

Anyway, it is important to not only understand the impacts of domestic livestock grazing and biodiversity but also to include ecological, economic, and social perspectives on this issue (Kok et al., 2020).

## Case Study

Taking advantage of a pilot monitoring trial on integrated wildlife monitoring in Spain (Barroso et al., 2023), we evaluated differences in community composition, relative abundance, and biodiversity indexes (species richness and Shannon diversity index) in areas with (*n* = 8) and without (*n* = 4) livestock presence. Twelve sites were selected for being representative of mainland Spain in terms of habitats, climate, and livestock management systems. A grid of 20 camera traps (Browning Strike Force HD ProX, Browning Arms Company, USA) was deployed for 40 days in each study site and the survey effort per area was harmonized to 339 camera-days (range: 222-358 camera-days). The relative abundance of livestock species, birds and small mammals was calculated as the sum of their trapping rates, i.e., the number of contacts of each species divided by the number of sequences of the species by the total number of operative days (O’Brien, 2011). The relative abundance of livestock species represented the grazing intensity. Species richness was the number of different species detected by camera traps at each sampling point. Biodiversity was estimated by the Shannon diversity index (H) for the whole set of species (30 mammals and 50 bird species) as well as for wildlife species including birds (24 mammal and 50 bird species) and exclusively for birds. These metrics were computed using *ggvegan* R package.

**Figure 2.**
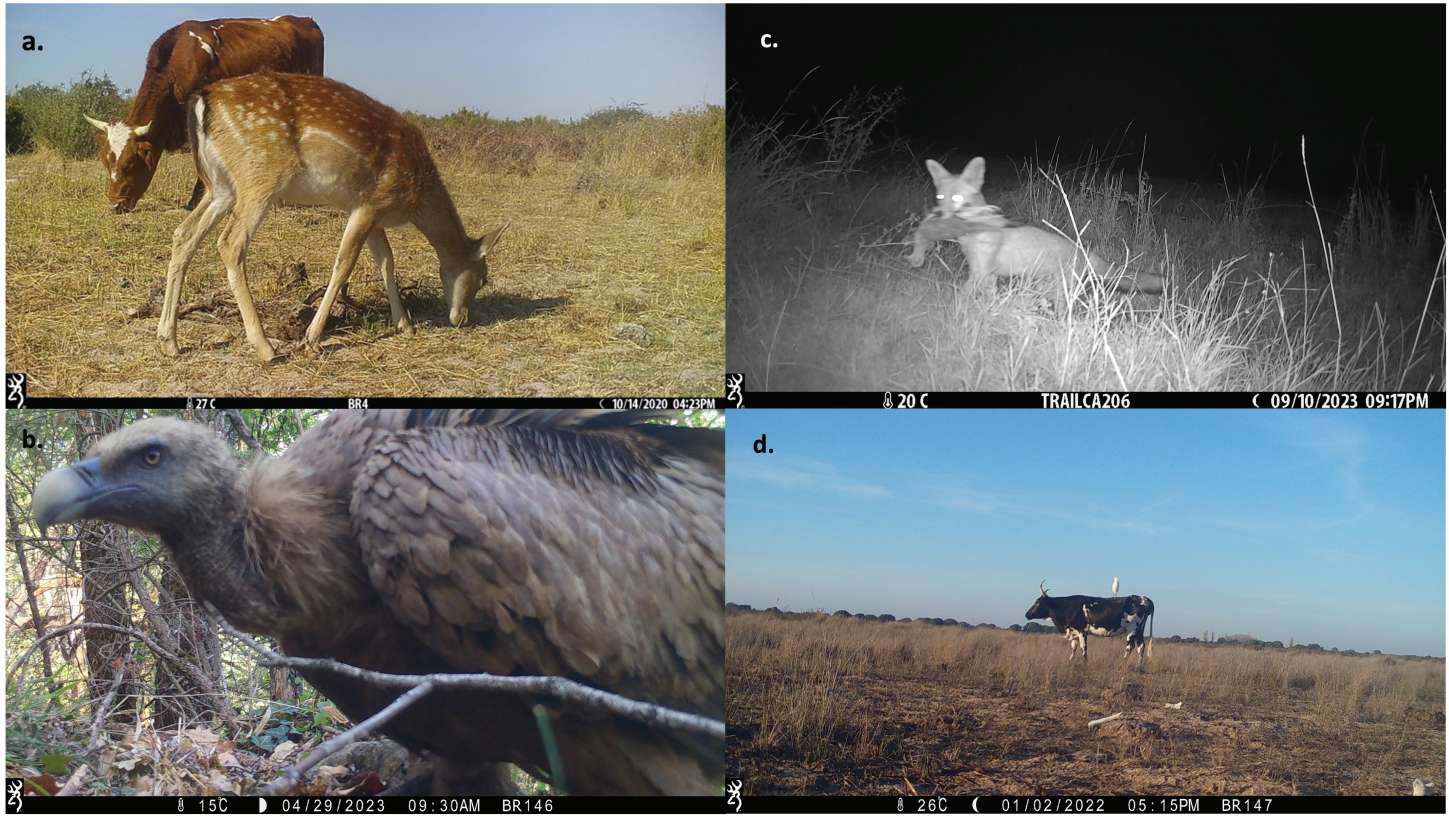
Images taken from camera traps in the context of a pilot monitoring trial for integrated wildlife surveillance in twelve sampling points from mainland Spain. (a) Direct interaction between a wild ungulate and cattle while sharing pastures with risk to pathogen transmission in both directions; (b) the presence of livestock could both benefit and impact scavenger populations by the higher availability of carcasses in the field and the indirect poisoning and exposure to veterinary drugs, respectively; (c) Wild carnivores feed mainly on birds and small mammals whose habitat suitability may be damaged by the livestock presence, which ultimately may lead to a decline in their population; (d) some species such as the cattle egret (*Bubulcus ibis*) have historically co-evolved with grazing livestock species.

An important issue that arises from the images captured by camera traps is the cumulative grazing pressure (Neilly et al., 2016). Thus, not only cattle are responsible for changes and habitat degradation for wildlife but also other wild herbivores with which they share pastures, also representing a risk for the transmission of shared pathogens in both directions as can be observed in Figure 2a. Figure 2b shows the presence of vultures (*Gyps fulvus*) in livestock areas where they could be partly favored, as extensive livestock is a key source of carrion, but also harmed by exposure to poisons and drugs (Aguilera-Alcalá et al., 2022). Figure 2c and d displays predation or mutualism relationships between species.

**Figure 3.**
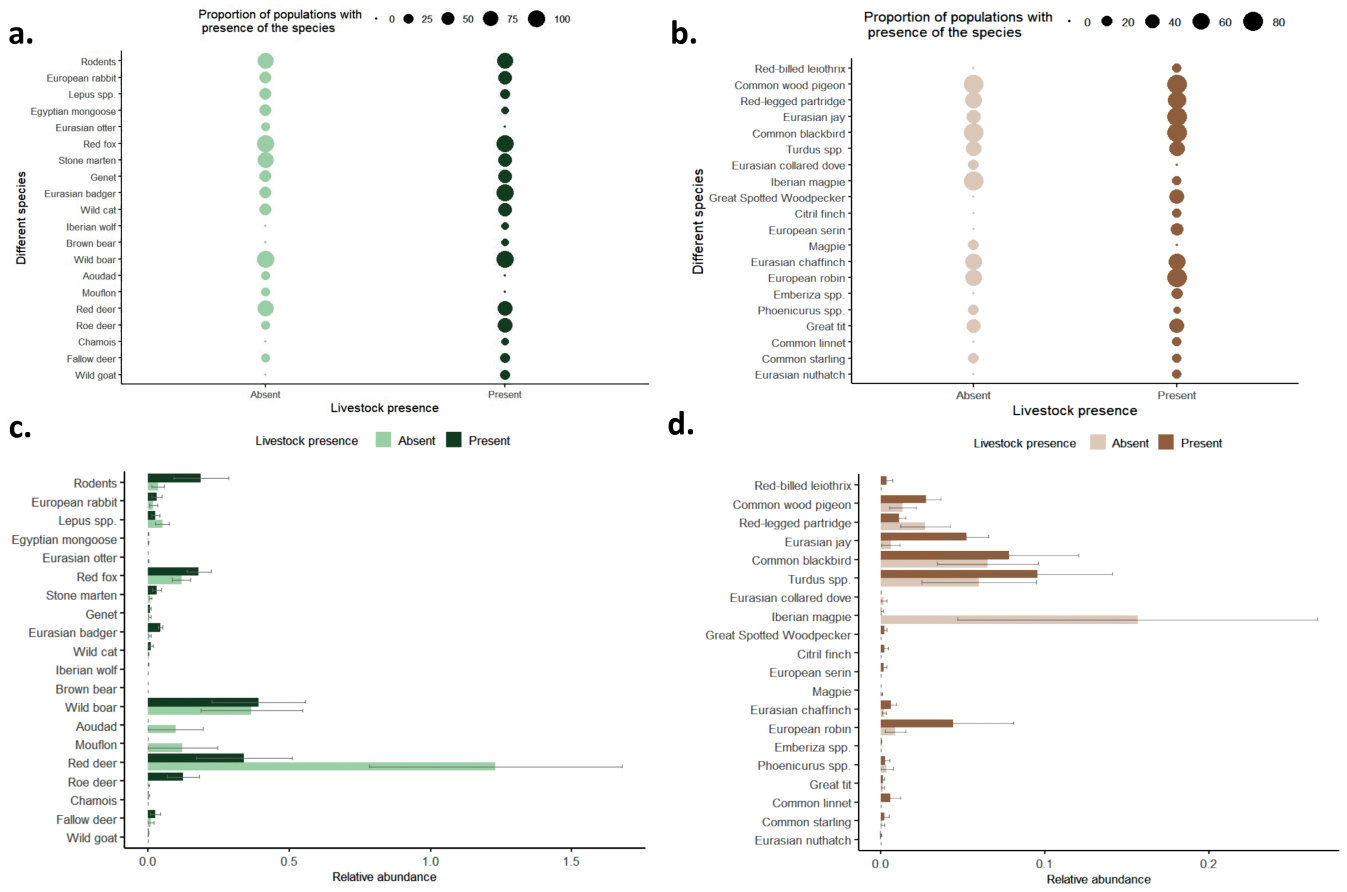
Results from camera traps in the context of a pilot monitoring trial for integrated wildlife surveillance in 12 sampling points from mainland Spain. Bubble plot displaying the proportion of study sites in which (a) mammal and (b) bird species were present depending on the livestock presence. The barplots indicate the average (±standard error) trapping rate of each species ((c) mammals and (d) bird species) in those study sites where they were present.

Figure 3 represents the species composition of the study sites depending on the livestock presence separately for mammals and birds. The most relatively abundant species were included in these graphs. Generalist species such as wild boar (*Sus scrofa*) and red fox (*Vulpes vulpes*) were recorded at all sampling sites, and at very similar relative abundances. The relative abundance of rodents was significantly higher in areas with livestock presence (Kruskal-Wallis X^2^: 4.03; *p*: 0.04) and was directly correlated with the relative abundance of livestock (R: 0.60; *p*: 0.05).

**Figure 4.**
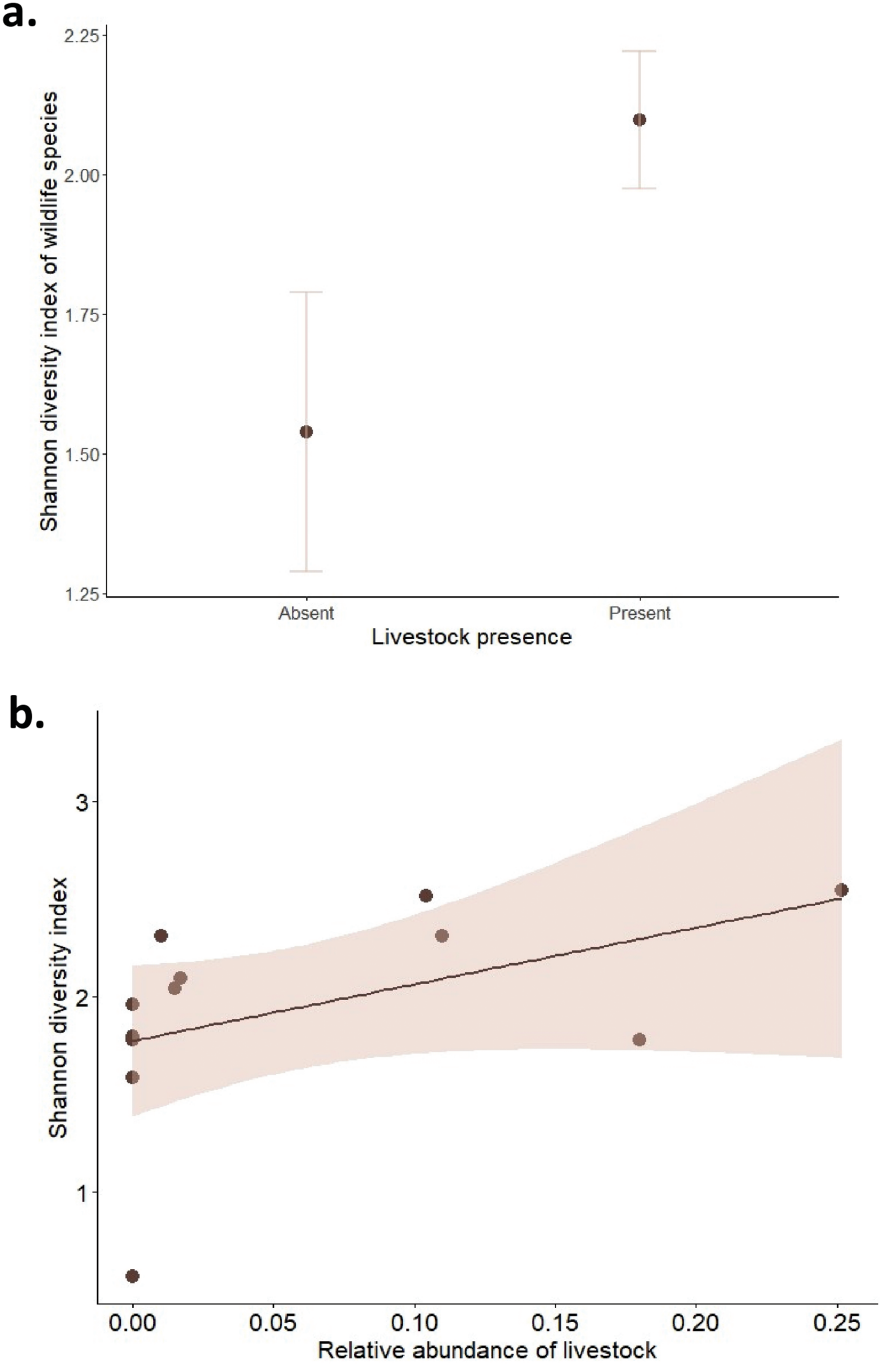
Results from camera traps in the context of a pilot monitoring trial for integrated wildlife surveillance in 12 sampling points from mainland Spain. (a) Biodiversity calculated as the Shannon diversity index (H) of wildlife species depending on the livestock presence. (b) Biodiversity calculated as the Shannon diversity index (H) depending on the relative abundance of livestock species (number of sequences per camera trap and day including cattle, horse, sheep, and goat).

In general, there was a statistically significant higher species richness and biodiversity in areas with livestock (*p* < 0.05; Figure 4a). Interestingly, the biodiversity increased with the relative abundance of livestock grazing species, but the maximum relative abundances found were considered as moderate (Figure 4b) (Rosenthal et al., 2012). Thus, the effect of heavy grazing could not be evaluated. Finally, during this trial, we also noted the conflict of livestock presence in protected areas. There are several regions across the globe where livestock grazing within protected areas has been allowed historically such as Currituck National Wildlife Refuge in North Carolina, the Alpine National Park in Australia or Doñana National Park in Spain (Porter et al., 2014; Williamson et al., 2014; Barroso et al., 2020). These situations highlight the interdependence between biodiversity, pastoral farming and socio-economic since excluding livestock from these areas can be expensive and socially controversial (Neilly et al., 2016; Norton et al., 2020). In this context, understanding the bidirectional effects of biodiversity-livestock interface is crucial to better manage these areas.

## Win–win outcomes for livestock–biodiversity interface

To encompass livestock profitability and the achieving of conservation goals it is necessary to look at more moderate grazing, considering that both livestock and native wild herbivores species may contribute to habitat degradation (Neilly et al., 2016; Kok et al., 2020). In agroecosystems, conservation could be performed through land sparing (i.e., exclude livestock production from certain lands to its restoration for conservation purposes) or land sharing perspectives (i.e., combine biodiversity conservation with compatible livestock production) (Fischer et al., 2017; Gordon, 2018). Land sharing implies some actions aimed at enhancing biodiversity by minimizing grazing impacts on rangelands (shrub retention, planting native trees, avoiding erosion, rotational grazing, reducing nutrient losses by targeting animal type and fertilizers use, etc.) and ensuring food and shelter supplies for wildlife (Santos-Martín et al., 2019; Norton et al., 2020).

**Figure 5.**
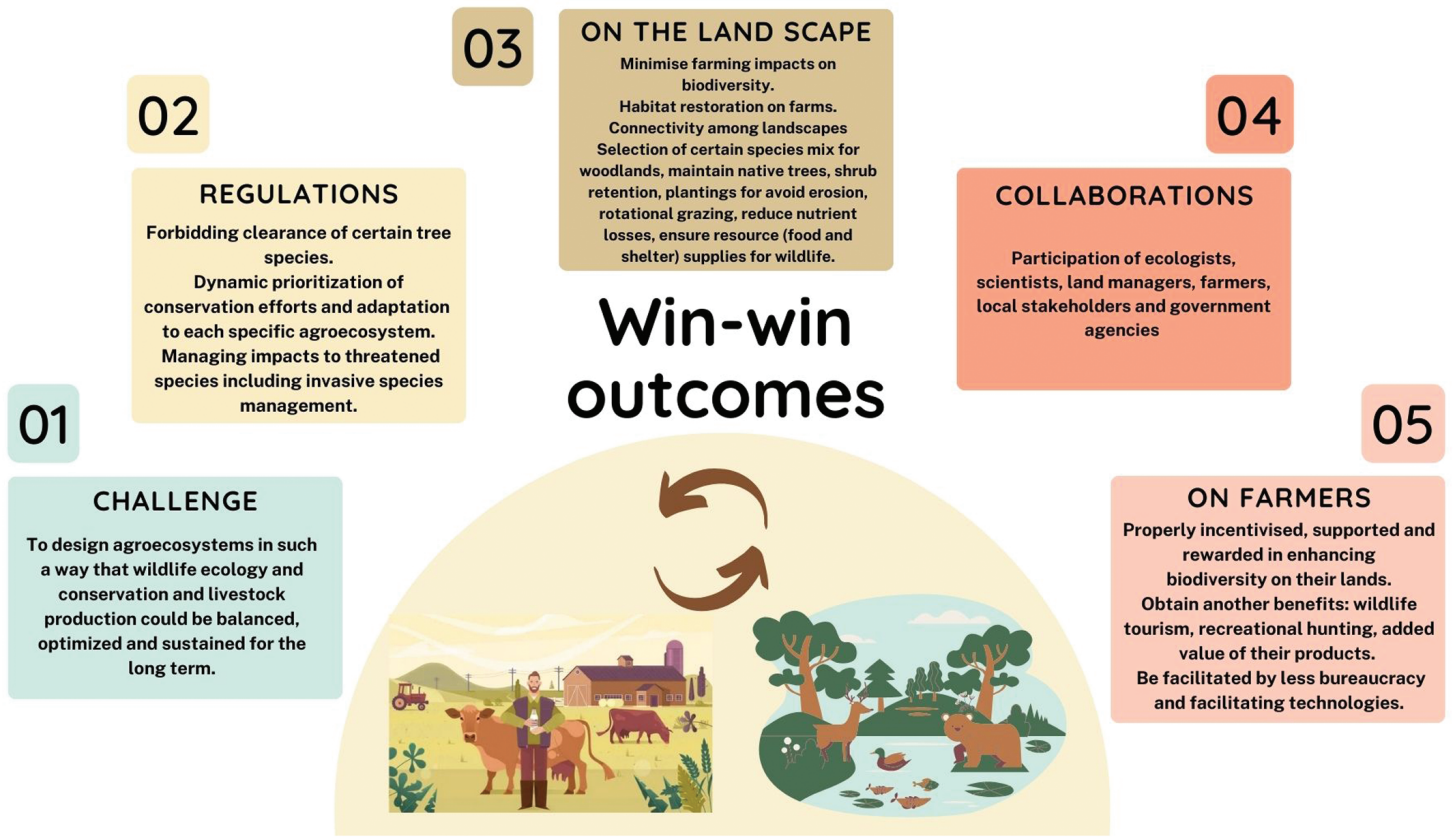
Factors involved in reaching sustainable win-win outcomes in the biodiversity-livestock interface.

However, the influence of the economic and social sphere on the biodiversity-livestock interface should not be neglected (Fischer et al., 2017). In this regard, reaching sustainable win-win outcomes becomes essential (Figure 5) (Santos-Martín et al., 2019; Norton et al., 2020). The landowners could become the best managers of biodiversity on their land, and they should be properly valued, incentivized, and rewarded for biodiversity preservation instead of being subjected to strict regulations on livestock management (Norton et al., 2020). To make the livestock-biodiversity relationship economically viable in agroecosystems, obtaining other benefits such as producing value-added products, commercial hunting, or wildlife tourism is key (by accommodation and recreational opportunities for landowners) (Norton et al., 2020; Pozo et al., 2021).

Finally, rural activities should be satisfactory, viable and profitable for farmers and landowners (e.g., by less bureaucracy when applying for agricultural and rural development support or facilitating technologies, among other interventions) to reach socially sustainable rural development (Granvik et al., 2012).

## Conclusion

Balancing biodiversity and livestock production outcomes in agroecosystems is a must (Neilly et al., 2016). Our capability to manage biodiversity conservation on areas with a wildlife-livestock interface, including protected areas and rangelands, is driven by the understanding of its bidirectional consequences. Cooperation among ecologists, scientists, land managers, farmers, and government agencies is one of the main tools available to achieve the desired balance between food production industries and wildlife ecology and conservation (Treves et al., 2006). The aim should not be only to increase the richness and diversity of wild species in territories with livestock but also to dynamically prioritize efforts aimed at conservation and adapt them to each specific system (Pozo et al., 2021). There is a high interconnection between biodiversity conservation and livestock production in which win-win outcomes are plausible for both relevant actors. For this purpose, landowners could exert an essential role in conserving and enhancing biodiversity since they are the best managers of native wildlife on a local scale. The challenge to achieve this objective is to design agroecosystems in such a way that biodiversity and livestock productivity can be optimized and sustained for the long term. Thus, instead of landowners being subject to strict regulations, they should be facilitated, encouraging their active involvement in biodiversity preservation.

## About the Authors

**Dr. Patricia Barroso** is a researcher for the Department of Veterinary Sciences of the University of Turin. Main research activities include health and population monitoring of wildlife, eco-epidemiology of shared pathogens at the wildlife-livestock-human interface, and disease control. Main research topics include the long-term dynamics and epidemiology of shared pathogens in complex host communities, the design of integrated wildlife monitoring systems and the study of biodiversity-disease relationships.

**Dr. Stefania Zanet** received her Veterinary Degree from the University of Turin, Italy in 2009. From the same University, she received a PhD in Natural Sciences and Innovative Technologies in 2014. Her main research interests are vector-borne diseases, especially the aspects regarding alternative wild reservoir species and livestock-wildlife interactions in disease epidemiology. She is an author for numerous peer-reviewed scientific publications and is currently member of the ECZM-WHP Examination Committee.
